# 

**Published:** 1887-07

**Authors:** 


PAGE.
ORIGINAL COMMUNICATIONS—A Critical Comparison of a Series of Skulls of the Wild and Domesticated Turkey. R. W. Shufeldt, M.D. ........ 207
"Surra," or Progressive Pernicious Anaemia. R. W. Burke, A.V.D ........ 223
Aneurism of the Mesenteric Artery. R. W. Burke, A.V.D ........ 230
Technics of Animal Vaccination. W. L. Zuill, M.D., D. V. S. ........ 233
Some Familiar Practical Methods in Bacteriology. E. O. Shakespeare, A.M., M.D ........ 241
Discolation of the Eye-ball in Dogs. Daniel D. Lee, M.D.V ........ 246
The Cause of Influenza-pectoralis of Horses. Johann W. Schutz, M. D ........ 248
D. E. Salmon, with Portrait ........ 257
EDITORIAL DEPARTMENT ........ 262
CASE DEPARTMENT ........ 267
SELECTIONS FROM FOREIGN PERIODICALS ........ 271
PROCEEDINGS OF VETERINARY MEDICAL SOCIETIES ........ 280
OBITUARY ........ 286
REVIEWS ........ 288
PROGRESS OF VETERINARY SCIENCE ........ 293

